# Health seeking behavior and self-medication practice among undergraduate medical students of a teaching hospital: A cross-sectional study

**DOI:** 10.1016/j.amsu.2022.103776

**Published:** 2022-05-13

**Authors:** Sitaram Khadka, Oshan Shrestha, Gaurab Koirala, Utshab Acharya, Gopal Adhikari

**Affiliations:** a Department of Pharmacy, Shree Birendra Hospital, Nepalese Army Institute of Health Sciences, Kathmandu, 44600, Nepal; b College of Medicine, Nepalese Army Institute of Health Sciences, Kathmandu, 44600, Nepal

**Keywords:** Students, Medical, Patient Acceptance of Health Care, Socioeconomic Factors, Prescriptions

## Abstract

**Introduction:**

Health seeking behavior is any activity undertaken by individuals who find themselves with a health-related problem with the aim of seeking an appropriate remedy. It includes decision making that is not homogenous among all as it is determined by different demographic factors. Self-medication and alternative medicine are also choices made by an individual that comes under health care seeking behavior. This study aimed to put light on the health seeking behavior of undergraduate medical students and to assess how they deal with their illnesses, including the practise of self-medication.

**Methods:**

In this web-based cross-sectional study, conducted among undergraduate medical students, a total of 210 students were selected through a systematic sampling method. The data were analyzed using SPSS version 20. Frequency was calculated for all the variables. The Mann-Whitney U or Kruskal-Wallis H test was used to see if any difference existed in the response. A significant association was declared at a p-value less than 0.05.

**Results:**

Response from 208 respondents was received and among those 88.9% of the respondents were aware of physical, mental and social aspects of health; about 40.8% of the respondents sought help only when their symptoms got worse; while 27.4% of them sought the help of alternative medicine. Around 74.2% of the respondents took medicines without a proper prescription and the commonly self-medicated drug group was NSAIDs.

**Conclusions:**

The knowledge of medical science has not satisfactorily ensured better health-seeking behavior and good practices. Also, there is high prevalence of self-medication practice among medical students.

## Highlights

• Health seeking behavior is activity of individuals to seek an appropriate remedy.
• Decision making of seeking help is not homogenous among all.
• Health seeking behavior is determined by different demographic factors.
• Knowledge of medical science only has not ensured better health-seeking behavior.
• Practice of self-medication has added another burden.

## 1 Introduction

Health seeking behavior is often understood as any activity undertaken by individuals who find themselves with a health-related problem or illness with the aim of seeking an appropriate remedy [1]. It is anteceded by a decision-making process that is further governed by individual and/or household behavior, community norms, and expectations as well as provider related characteristics and behavior [2]. It is not homogenous as it is determined by the factor of cognition or awareness, socio-cultural as well as economic factors. The interplay of these factors plays a crucial role in the decision making by an individual [2]. Seeking help of alternative medicine and self-medication is also a choice made by an individual that comes under health care seeking behavior [3]. This act differs from self-care as it involves drugs that have the possibility to do good or cause harm [4]. As medical students have knowledge of both drugs and illnesses, they are more prone to this practice.

Health seeking behavior and decision making regarding his/her health is determined different factors including individual and/or household behavior, community norms, and expectations. This study aims to find out the awareness of medical students on all these aspects regarding their own health and also to find out the prevalence of self-medication practice among medical students.

## 2 Materials and methods

### 2.1 Ethical consideration

The research has been performed following the Declaration of Helsinki. The ethics approval was obtained from the Institutional Review Committee of the Nepalese Army Institute of Health Sciences (NAIHS-IRC) (Ref no: 485). Information regarding the nature of the study and consent form were included in the questionnaire itself. All the participants were made aware that their participation was voluntary and they were assured of confidentiality prior to the data collection.

### 2.2 Study design and setting

This cross-sectional study was donefrom 22nd December 2021 to 27th January 2022, among the undergraduate medical students of a teaching hospital to assess their health seeking behavior and attitude towards self-medication. The study took place at Nepalese Army Institute of Health Sciences- College of Medicine (NAIHS- COM) which is located in the capital city of the country and where students from all corners of the country visit for higher education. Students of the second year to the fifth year of medical school were included in this study. Students of the first year couldn't be included as it was the time of the new academic session and they were not yet admitted to the college. A list of students from the second to the fifth year was accessed and respondents were selected from the list by following the systematic sampling method. Only the selected ones were sent the web link of “Google Form” containing the questionnaire through email. Each respondent was asked for their consent and only those who gave the consent were allowed to go to the next page of the data collection form. This study is in line with STROCSS 2021 criteria [5].

### 2.3 Sample size

We used Cochran's formula [6] to get the target sample size.

n = Z^2^ × p × q **/**e^2^

= 1.96^2^ × 0.5 × 0.5/ 0.05^2^

= 384

where.

n = calculated sample size
Z = 1.96 at 95% Confidence Interval
p = expected prevalence of students who have poor health seeking behavior, 50%
q = 1-p
e = Margin of error (5%)

Number of undergraduate medical students from second to fifth year of medical school (N): 415

Adjusted sample size = n / (1+ n/N)

= 384/ (1 + 384/415)

= 200

Considering a 5% non-response rate, the final sample size was 210.

### 2.4 Sampling technique

A systematic sampling method was used. The list of students (from the second year to final year) was accessed and every 9th (computer-generated random number) person on the list was choosen as our responder.

### 2.5 Study tool

A questionnaire was prepared after a thorough literature review to include questions related to physical, social, and mental dimensions of health. A questionnaire with demographic questions and twenty Likert items was prepared for this study. This study tool assessed the knowledge, attitude, and practice of the medical students towards health attention seeking and self-medication practice. Study tool is available as Supplementary File 1.

### 2.6 Analytical strategy

Individual Likert items of the questionnaire were treated as an ordinal variable and non-parametric tests were utilized. Likert items were not grouped to produce a single result, instead, each Likert item was analyzed separately. Data was analyzed using Statistical Package For The Social Sciences (SPSS) version 20. Frequency was calculated for all the independent variables and dependent variables. Since our dependent variables were ordinal and independent variables were categorical, the Mann-Whitney *U* test or Kruskal-Wallis H test was used as per fit to see if any difference existed in the response of the responders concerning different categories of independent variables [7]. In the text, when mentioning the percentage of responders that agreed to a particular question asked frequency of Strongly Agree and Agree responses were added. Similar was done while mentioning the frequency of responders that disagreed (addition of Strongly Disagree and Disagree). In the table, the frequency was given on a five-point scale (Strongly Disagree, Disagree, Neutral, Agree, and Strongly Agree).

## 3 Results

Out of 210 selected students, this study had 208 respondents, out of which 151 (72.6%) were male and 57 (27.4%) were female. The age of the participants ranged from 17 to 26 years with a mean age of 22.17 ± 1.43 years (Mean ± SD). The responders were from all the provinces and various ethnic groups, while the majority were from Bagmati province and Chhetri ethnicity. Details of other socio-demographic characteristics of the respondents are listed in Table 1.

**Table 1:** Characteristics of the respondents.

Characteristics	Sample group (N = 208)	
	n	%
**Age (in years)**		
Late teen (17–19)	3	1.44
Early twenties (20–23)	163	78.36
Mid-twenties (24–26)	42	20.19
**Sex**		
Male	151	72.6
Female	57	27.4
Year of Study		
Pre-Clinical	58	27.9
Clinical	150	72.1
**Permanent address**		
Province 1	11	5.3
Madhesh	38	18.3
Bagmati	86	41.3
Gandaki	36	17.3
Lumbini	27	13.0
Karnali	3	1.4
Sudurpaschim	7	3.4
**Daily exercise/yoga/outdoor sports**		
Yes	88	42.3
No	120	57.7
**Ethnicity**		
Chhetri	75	36.1
Brahmin	72	34.6
Magar	6	2.9
Tharu	5	2.4
Newar	11	5.3
Others	39	18.8
**Have strong faith in god**		
Yes	176	84.6
No	32	15.4
**Socio-economic condition of the family**		
Lower class	2	1.0
Working class	9	4.3
Lower middle	74	35.6
Upper middle	114	54.8
Upper class	9	4.3
**Highest level of education attained by either parent**		
Informal education	9	4.3
High school	37	17.8
University level	162	77.9

More than two-thirds of the responders (88.9%) Were mindful about the different aspects of health (physical, social and mental). About 75.5% of the responders sought help immediately when they developed some physical symptoms whereas, 40.8% agreed that they sought help only when their physical symptoms got worse. About 83.7% of the respondents pay equal attention to their physical and mental well-being and 27% felt that they neglect their social well-being.

About 38.9% of the medical students thought that their own college's hospital is good for them to seek help regarding their illness. While 42.8% of the students would opt for other hospitals to seek health attention regarding sensitive matters (taboo associated health conditions). The majority (52.9%) of them did not choose video-call with the physician (online consultation) as a substitute for in-person consultation with the physician. About a quarter of the respondents (27.4%) sought help from alternative medicine in addition to allopathy. The frequency of the responses given by the responders to other questions is shown in Table 2.

**Table 2:** Frequency of the responses.

Questions (Likert items)	Strongly Disagree n (%)	Disagree n (%)	Neutral n (%)	Agree n (%)	Strongly Agree n (%)
1. I am well aware that health includes physical, mental and social well-being and not just absence of disease/infirmity. (Q1)	16 (7.7)	0	7 (3.4)	61 (29.3)	124 (59.6)
2. I give equal attention to my mental well-being compared to physical well-being. (Q2)	10 (4.8)	5 (2.4)	19 (9.1)	106 (51.0)	68 (32.7)
3. I feel that I neglect my social well-being. (Q3)	21 (10.1)	86 (41.3)	45 (21.6)	49 (23.6)	7 (3.4)
4. I seek help immediately when I develop some physical symptoms. (Q4)	8 (3.8)	8 (3.8)	35 (16.8)	126 (60.6)	31 (14.9)
5. I only seek help when my symptoms get worse. (Q5)	12 (5.8)	75 (36.1)	36 (17.3)	71 (34.1)	14 (6.7)
6. I have faced difficulty in making/maintaining meaningful positive relationship with other people. (Q6)	28 (13.5)	83 (39.9)	36 (17.3)	53 (25.5)	8 (3.8)
7. I don't think that problems in making/maintaining meaningful positive relationship with other people are concerns of health. (Q7)	41 (19.7)	97 (46.6)	35 (16.8)	30 (14.4)	5 (2.4)
8. I believe that only weak and unproductive people are bothered by problems of mental and social well-being. (Q8)	66 (31.7)	103 (49.5)	18 (8.7)	17 (8.2)	4 (1.9)
9. I don't think that problems of thoughts, feelings and coping ability with ups and down of life are concerns of health. (Q9)	58 (27.9)	95 (45.7)	19 (9.1)	32 (15.4)	4 (1.9)
10. I am willing to talk to my friends and family if I face problems of thoughts, feelings and coping ability. (Q10)	6 (2.9)	14 (6.7)	27 (13.0)	121 (58.2)	40 (19.2)
11. My own college's hospital is best for me to seek help for my health problems. (Q11)	18 (8.7)	45 (21.6)	64 (30.8)	76 (36.5)	5 (2.4)
12. I prefer going to hospitals other than my own college's hospital to seek help for sensitive matters (taboo associated health conditions). (Q12)	5 (2.4)	41 (19.7)	73 (35.1)	72 (34.6)	17 (8.2)
13. I don't think public health care facilities of Nepal provide standard care. (Q13)	7 (3.4)	38 (18.3)	51 (24.5)	95 (45.7)	17 (8.2)
14. In my opinion only private health care facilities provide standard care in Nepal. (Q14)	15 (7.2)	71 (34.1)	52 (25.0)	61 (29.3)	9 (4.3)
15. I would opt for online-consultation over a video call with the physician as a substitute to in-person consultation. (Q15)	36 (17.3)	74 (35.6)	56 (26.9)	38 (18.3)	4 (1.9)
16. I seek for alternative medicine in addition to allopathic medicine. (Q16)	25 (12.0)	61 (29.3)	65 (31.3)	53 (25.5)	4 (1.9)
17. I try to find out about my illness on my own before going to a doctor. (Q17)	3 (1.4)	13 (6.3)	39 (18.8)	132 (63.5)	21 (10.1)
18. I sometimes take medicine without prescription when I am sure about my illness. (Q18)	11 (5.3)	32 (15.4)	29 (13.9)	119 (57.2)	17 (8.2)
19. I suggest my friends and family to take certain medicines when they ask for suggestions. (Q19)	16 (7.7)	49 (23.6)	51 (24.5)	87 (41.8)	5 (2.4)
20. It is fine to take the medicine by myself when I am sure about the medical condition. (Q20)	13 (6.3)	53 (25.5)	46 (22.1)	89 (42.8)	7 (3.4)

Abbreviation: Q: Question.

Likert items were subjected to inferential statistics to see if there was a difference in responses in relation to the different categories of independent variables. It was seen that responses to Q8, Q12, Q19, and Q20 differed significantly between male and female whereas responses to Q17, Q18, Q19, and Q20 differed significantly between pre-clinical and clinical students. Similarly, the responses to Q3 and Q6 differed significantly among those who were involved in daily exercise/yoga/outdoor sports and those who were not while responses to Q3 and Q14 differed significantly between students that kept strong faith in God and those who did not. Likewise, the difference in responses to Q3, Q7, Q8, and Q17 was significant among socio-economic strata and responses to Q3 and Q7 differed significantly among the different level education attained by either parent of the students. Statistically significant analyses are summarized in Table 3 and details of all the analyses are available as Supplementary File 2.

**Table 3:** Result of different tests.

Analyses	P-value	Test Used
I feel that I neglect my social well-being (Q3) * Daily exercise/yoga/sports	0.0003	Mann-Whitney U
Q3 * Strong faith in god	0.01	Mann-Whitney U
Q3 * Socio-economic level of family	0.00003	Kruskal-Wallis H
Q3 * Highest education level of either parent	0.02	Kruskal-Wallis H

I have faced difficulty in making/maintaining meaningful positive relationships with other people (Q6) * Daily exercise/yoga/sports	0.003	Mann-Whitney U

I don't think that problems in making/maintaining meaningful positive relationship with other people are concerns of health (Q7) * Socio-economic level of family	0.01	Kruskal-Wallis H
Q7 * Highest education level of either parent	0.002	Kruskal-Wallis H

I believe that only weak and unproductive people are bothered by problems of mental and social well-being (Q8) * Sex	0.02	Mann-Whitney U
Q8 * Socio-economic level of family	0.003	

I prefer going to hospitals other than my own college's hospital to seek help for sensitive matters (Q12) * Sex	0.02	Mann-Whitney U

In my opinion, only private health care facilities provide standard care in Nepal (Q14) * Strong faith in god	0.02	Mann-Whitney U

I try to find out about my illness on my own before going to a doctor (Q17) * Study Year	0.02	Mann-Whitney U
Q17 * Socio-economic level of family	0.04	Kruskal-Wallis H

I sometimes take medicine without prescription when I am sure about my illness (Q18) * Study Year	0.01	Mann-Whitney U

I suggest my friends and family to take certain medicines when they ask for suggestions (Q19) * Sex	0.02	Mann-Whitney U
Q19 * Study Year	0.00006	Mann-Whitney U

It is fine to take the medicine by myself when I am sure about the medical condition (Q20) * Sex	0.002	Mann-Whitney U
Q20 * Study Year	0.04	Mann-Whitney U

Abbreviation: Q: Question.

**Fig. 1:**
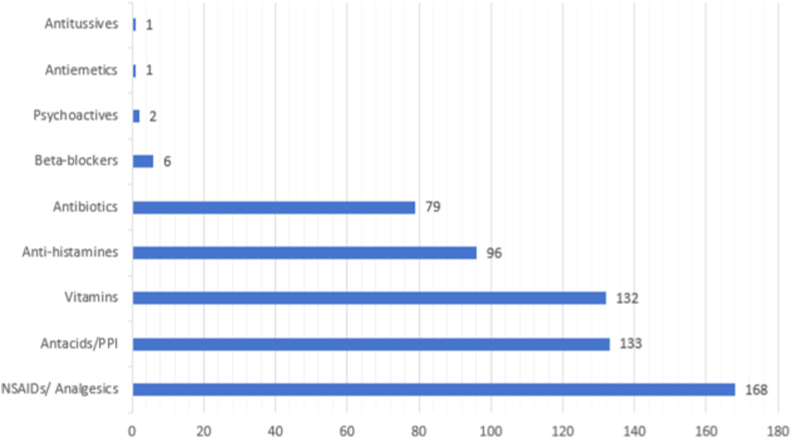
Commonly self-medicated drugs.

About 74.2% of the responders agreed that they have taken medicines without a proper prescription, 46.2% of the responders considered self-medication a fine practice if one is sure of the medical condition and 44.2% of the responders suggested their family/friends to take certain medicines. The most commonly self-medicated drug among the respondents was non-steroidal anti-inflammatory drugs (NSAIDs) followed by antacids/proton-pump inhibitors (PPI). Antitussives, antiemetics, and psychoactive drugs were in the least frequency. Fig. 1 shows the frequency of commonly self-medicated drugs.

**Fig. 2:**
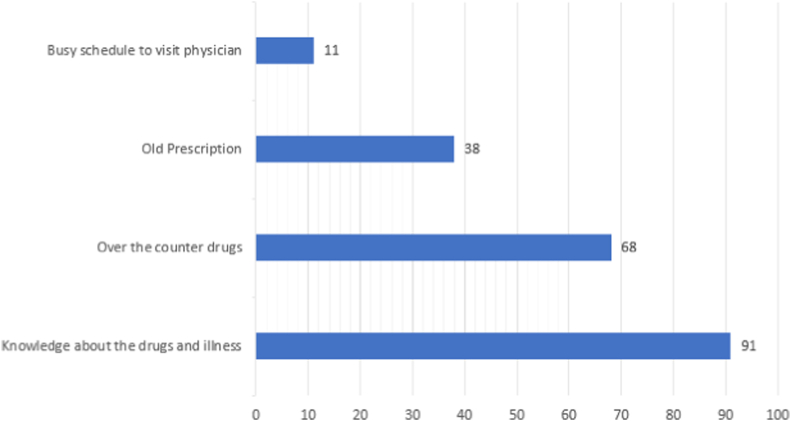
Reasons for self-medication.

The most common reason for self-medication was knowledge of the drug and the medical condition. Some also practiced self-medication based on old prescriptions. Fig. 2 shows the reasons for self-medication given by the responders.

## 4 Discussion

In this study, we focused on the realization of problems in different aspects of health, timing chosen by the patients to go for a check-up, mode of consultation, seeking the help of alternative medicine, and self-medication. This study is different from other studies as this has explored health-seeking behavior by taking the context of mental and social factors in addition to the physical aspect of health, as health is just not about physical well-being or just mental well-being, unlike other studies [8,9] which have focused on a specific disease/condition.

This study conducted among undergraduate medical students of a teaching hospital demonstrated that 88.9% of the responders were well known about the physical, mental, and social aspects of health. Still, 40.8% sought help only when their physical symptoms got worse, 17.3% of the responders didn't consider problems of thoughts, feelings, and coping ability with ups and downs of life as concerns of health, and 16.8% of them didn't perceive problems in making/maintaining a meaningful positive relationship with other people as concerns of health. Despite having the same academic backgrounds, still, 10.1% of responders believed that only weak and unproductive people suffer from issues of mental and social well-being. It is to be noted that respondents of this study are currently being trained to be health professionals under the same curriculum. The only difference prevailing among the responders is the socio-economic variables. And, this has brought this disparity among respondents as social, economic, and legal variables affect the way health and disease are perceived by an individual [10].

Talking about the ailing state of social health of the students, this may be linked to the nature of their study and the time demanded by their course which restricts their social life. Since medical education is a long and demanding course, medical students are prone to get distressed which can lead to decreased cognitive functioning and depression [11]. Medical students are vulnerable to all these compared to others of the same age group [12]. Our study found out that 27% of the students felt that they neglect their social well-being, while 16.8% of the respondents were unaware that the problems in making/maintaining a meaningful positive relationship with other people are concerns of health.

About 42.8% of the respondents who sought help for sensitive matters and taboo associated diseases chose other hospitals/clinics than their own college's hospital. This indicates their concern towards confidentiality and stigma. A previous study pointed out that the students are concerned that seeking mental health care would raise questions among colleagues and faculties regarding their stability and appropriateness that is demanded by the medical fraternity [13]. Besides this, medical students face different barriers while seeking attention. The fear of stigma, issues of confidentiality, confusion on seeking help, fear of unwanted intervention while seeking mental healthcare, and barriers like monetary issues, lack of time, and fear of side effects while seeking physical healthcare are such identified barriers [14]. Renee et al. have also mentioned in their study that medical centers outside the university offer students sufficient distance for them to feel assured of their confidentiality [15].

All these findings just point out the same thing, no matter what the current academic qualifications, training, and level of awareness are, there will be socio-cultural determinants that affect the state of health. People tend to blend in the concepts of health, disease, and healthcare-seeking behavior according to their lifestyles and their respective demographic circumstances, such that, knowledge of medicine alone cannot ensure better healthcare-seeking behavior [16]. The findings of our study are in line with this. Health-seeking behavior is a very complex matter as what is defined as health or infirmary varies with cultures, life experiences, socio-economic variables, and legal variables [10,17]. Healthcare seeking behavior differs from person to person as it is a very personal matter [18] and healthcare involves interaction with other individuals [19].

Since the responders of this study were medical students, 73.6% of them were found to investigate their illnesses on their own before going to a doctor and 65.4% of the students were found to be taking medicine on their own when they were sure about their diagnosis. Given the knowledge of medical students regarding various diseases and drugs, this practice is common among medical students and studies also have shown a prevalence of self-medication from 44.8% [4] to a very high prevalence of 92% [3]. Self-medication practice in this study was found to differ significantly with study year and a similar result was observed in a study where prevalence increased from first to final year [20]. This study found that over-the-counter (OTC) drugs; NSAIDs/analgesics, and antacids/PPIs were most commonly used in self-medication practice. NSAIDs/analgesics being the most common is consistent with the findings of other studies done among foreign medical students [[20], [21], [22]]. However, self-medication should not be confused with self-care and as a safe way of healthcare utilization. Self-medication is a dangerous practice and has its risks involved which outweigh its few benefits like quick relief. Its risks can involve the wrong manner and route of administration and may lead to adverse reactions, serious drug interactions, masking-off of severe illness, dependence, and abuse [23].

During the coronavirus disease (COVID-19) pandemic, the mode of consultation of the patients has also switched to online in substitute to in-person consultation [24]. Patients have been looking for an alternative to face-to-face consultation, and online as well as telecommunication-based consultations are in practice [[25], [26], [27]]. More than half of the responders disregarded online consultation as a substitute for in-person consultation with the physician. This might be attributed to their nature of study and their accessibility to physicians.

## 5 Conclusion

Health-seeking behavior is a complex and very personal matter of an individual which is mostly determined by their demographic circumstances. The knowledge of medical science has not satisfactorily ensured better health-seeking behavior and good practices. The high prevalence of practice of self-medication adds another burden. There is a need for timely and evidence-based teaching-learning techniques; consideration of individual and/or household behavior, community norms, and expectations for holistic approach; and executing legislation to reduce the barriers and upgrade the health seeking behavior of medical students.

Recommendations: Undergraduate medical students are not well qualified to have better judgment of their well-being and the practice of self-medication may pose a significant risk with unwanted consequences. In this regard, the findings from the present study demand special attention to educating undergraduate medical students through timely and evidence-based teaching-learning techniques on the types of illnesses that can be self-diagnosed and self-treated and the types of drugs to be used for self-medication. Equal focus should be given to the different factors including individual and/or household behavior, community norms, and expectation that determine the health seeking behavior and self-medication practice. The respondents’ perspectives from the present study help policy-making bodies to prepare relevant public health strategies based on population-focused approaches to upgrade judicious and safe use of self-medication practices and health seeking behavior of medical students.

Limitations of the study: There are some limitations linked to this study. This study was carried out only in the institute located in Kathmandu, the capital city of Nepal. Therefore, the result obtained may not be generalized to the health seeking behavior of medical students all over the globe. However, the findings in other places did not vary much on literature review.

## Ethical approval

The research has been performed following the Declaration of Helsinki. The ethics approval was obtained from the Institutional Review Committee of the Nepalese Army Institute of Health Sciences (NAIHS-IRC) (Ref no: 485).

## Sources of funding

This article didn't receive any kind of grants.

## Author contribution

Sitaram Khadka (SK) and Oshan Shrestha (OS) were invoved in conceptualization of the study. OS, Gaurab Koirala (GK), Utshab Acharya (UA), and Gopal Adhikari (GA) were involved in data curation and drafting the initial version of the manuscript. OS and SK did the formal analysis and SK edited the manuscript intellectually. All the authors approved the final version of the manuscript.

## Registration of research studies

1. Name of the registry: Research Registry
2. Unique Identifying number or registration ID: researchregistry7788
3. Hyperlink to your specific registration (must be publicly accessible and will be checked): https://www.researchregistry.com/browse-the-registry#home/registrationdetails/624c4eec42081c001e95e7a7/

### Data availability statement

The curated data in this study is available through corresponding author on reasonable request.

## Guarantor

Oshan Shrestha.

College of medicine, Nepalese Army Institute of Health Sciences, Kathmandu-44600, Nepal.

Email: shresthaoshan93@gmail.com.

## Consent

Information regarding the nature of the study and consent form were included in the questionnaire itself. All the participants were made aware that their participation was voluntary and they were assured of confidentiality prior to the data collection.

## Peer review file

All authors agree to the publication of the peer review file for transparency.

## Provenance and peer review

Not commissioned, externally peer reviewed.

## Research registry

Registered in Research registry (researchregistry7788)

## Declaration of competing interest

No conflict of interest.
