# Supplementary material for: Health seeking behavior and self-medication practice among undergraduate medical students of a teaching hospital: A cross-sectional study
Source: Ann Med Surg (Lond). 2022 May 13;78:103776. doi: 10.1016/j.amsu.2022.103776 (PMC9117532; doi:10.1016/j.amsu.2022.103776)
Supplement: Multimedia component 2 [file mmc2.docx]

**Study Tool**

**Age (in years)**:

**Sex**: M/F

**Year of study**: Pre-clinical/Clinical

**Permanent Address** (Province): Province 1/ Madhesh/ Bagmati/ Gandaki/ Lumbini/ Karnali/ Sudurpaschim

**Daily exercise/yoga/outdoor sports**: Y/N

**Ethnicity**: Chhetri/ Brahmin/ Magar/ Tharu/ Newar/ Others

**Strong faith in god**: Y/N

**Socio-economic condition of the family**: Upper, upper-middle, lower-middle, working class, lower class

**Education of the parents** (highest attained by either parent): Elementary School, High School, University level, Informal Education

***Health Seeking Behaviour questionnaire:***

**Domain 1: Realization of health status and help seeking**

1. **I am well aware that health includes physical, mental and social well-being and not just absence of disease/infirmity.**

Strongly disagree

Disagree

Neutral

Agree

Strongly Agree

1. **I give equal attention to my mental well-being compared to physical well-being.**

Strongly disagree

Disagree

Neutral

Agree

Strongly Agree

1. **I feel that I neglect my social well-being.**

Strongly disagree

Disagree

Neutral

Agree

Strongly Agree

1. **I seek help immediately when I develop some physical symptoms.**

Strongly disagree

Disagree

Neutral

Agree

Strongly Agree

1. **I only seek help when my symptoms get worse.**

Strongly disagree

Disagree

Neutral

Agree

Strongly Agree

1. **I have faced difficulty in making/maintaining meaningful positive relationship with other people.**

Strongly disagree

Disagree

Neutral

Agree

Strongly Agree

1. **I don’t think that problems in making/maintaining meaningful positive relationship with other people are concerns of health.**

Strongly disagree

Disagree

Neutral

Agree

Strongly Agree

1. **I believe that only weak and unproductive people are bothered by problems of mental and social well-being.**

Strongly disagree

Disagree

Neutral

Agree

Strongly Agree

1. **I don’t think that problems of thoughts, feelings and coping ability with ups and down of life are concerns of health.**

Strongly disagree

Disagree

Neutral

Agree

Strongly Agree

1. **I am willing to talk to my friends and family if I face problems of thoughts, feelings and coping ability.**

Strongly disagree

Disagree

Neutral

Agree

Strongly Agree

**Domain 2: Preference of health facility**

1. **My own college’s hospital is best for me to seek help for my health problems.**

Strongly disagree

Disagree

Neutral

Agree

Strongly Agree

1. **I prefer going to hospitals other than my own college’s hospital to seek help for sensitive matters (taboo associated health conditions).**

Strongly disagree

Disagree

Neutral

Agree

Strongly Agree

1. **I don’t think public health care facilities of Nepal provide standard care.**

Strongly disagree

Disagree

Neutral

Agree

Strongly Agree

1. **In my opinion only private health care facilities provide standard care in Nepal.**

Strongly disagree

Disagree

Neutral

Agree

Strongly Agree

1. **I would opt for online-consultation over a video call with the physician as a substitute to in-person consultation.**

Strongly disagree

Disagree

Neutral

Agree

Strongly Agree

1. **I seek for alternative medicine in addition to allopathic medicine.**

Strongly disagree

Disagree

Neutral

Agree

Strongly Agree

**Domain 3: Self-diagnosing and self-medication**

1. **I try to find out about my illness on my own before going to a doctor.**

Strongly disagree

Disagree

Neutral

Agree

Strongly Agree

1. **I sometimes take medicine without prescription when I am sure about my illness.**

Strongly disagree

Disagree

Neutral

Agree

Strongly Agree

1. **I suggest my friends and family to take certain medicines when they ask for suggestions.**

Strongly disagree

Disagree

Neutral

Agree

Strongly Agree

1. **It is fine to take the medicine by myself when I am sure about the medical condition.**

Strongly disagree

Disagree

Neutral

Agree

Strongly Agree

**Domain 5**

1. **Reason for taking medicine without prescription?**
2. Old prescription
3. Knowledge about the drugs and illness
4. Over the counter drugs
5. Busy schedule to visit physician
6. Others
7. **Class of drugs used by you without prescription?**
8. Antibiotics
9. NSAIDS/ Analgesics
10. Vitamins
11. Anti-histamines
12. Antacids/PPI
13. Psychoactives
14. Tranquilizers
15. Antihypertensives
16. Beta- blockers
17. Others
