# Supplementary material for: Health seeking behavior and self-medication practice among undergraduate medical students of a teaching hospital: A cross-sectional study
Source: Ann Med Surg (Lond). 2022 May 13;78:103776. doi: 10.1016/j.amsu.2022.103776 (PMC9117532; doi:10.1016/j.amsu.2022.103776)
Supplement: Multimedia component 3 [file mmc3.docx]

| **Analyses** | **Mann-Whitney U**  **(Z value)** | **Chi- square (df)** | **P value** |
| --- | --- | --- | --- |
| **Q1 * Sex** | 4136.50 (-0.494) |  | 0.621 |
| **Q1 * Study Year** | 3866.50 (-1.42 ) |  | 0.15 |
| **Q1 * Permanent Address** |  | 4.33 (6) | 0.63 |
| **Q1 * Daily exercise/yoga/sports** | 5138.00 (-0.38 ) |  | 0.70 |
| **Q1 * Ethnicity** |  | 3.36 (5) | 0.64 |
| **Q1 * Strong faith in god** | 2509.50 (-1.12 ) |  | 0.26 |
| **Q1 * Socio-economic level of family** |  | 2.36 (4) | 0.67 |
| **Q1 * Highest education level of either parent** |  | 3.88 (2) | 0.14 |
|  |  |  |  |
| **Q2 * Sex** | 3896.00 (-1.154) |  | 0.249 |
| **Q2 * Study Year** | 4114.00 (-0.66 ) |  | 0.51 |
| **Q2 * Permanent Address** |  | 4.22 (6) | 0.65 |
| **Q2 * Daily exercise/yoga/sports** | 5080.50 (-0.51 ) |  | 0.61 |
| **Q2 * Ethnicity** |  | 3.96 (5) | 0.55 |
| **Q2 * Strong faith in god** | 2412.50 (-1.41) |  | 0.16 |
| **Q2 * Socio-economic level of family** |  | 1.01 (4) | 0.91 |
| **Q2 * Highest education level of either parent** |  | 1.67 (2) | 0.43 |
|  |  |  |  |
| **Q3 * Sex** | 3613.50 (-1.87 ) |  | 0.06 |
| **Q3 * Study Year** | 4112.50 (-0.64) |  | 0.52 |
| **Q3 * Permanent Address** |  | 2.99 (6) | 0.81 |
| **Q3 * Daily exercise/yoga/sports** | 3830.00 (-3.55 ) |  | 0.000379 |
| **Q3 * Ethnicity** |  | 7.91 (5) | 0.16 |
| **Q3 * Strong faith in god** | 2061.50 (-2.53 ) |  | 0.01 |
| **Q3 * Socio-economic level of family** |  | 25.69 (4) | 0.000037 |
| **Q3 * Highest education level of either parent** |  | 7.75 (2) | 0.02 |
|  |  |  |  |
| **Q4 * Sex** | 3906.50 (-1.17 ) |  | 0.24 |
| **Q4 * Study Year** | 4286.50 (-0.19 ) |  | 0.85 |
| **Q4 * Permanent Address** |  | 9.38 (6) | 0.15 |
| **Q4 * Daily exercise/yoga/sports** | 4944.50 (-0.89 ) |  | 0.37 |
| **Q4 * Ethnicity** |  | 3.56 (5) | 0.61 |
| **Q4 * Strong faith in god** | 2537.50 (-1.01 ) |  | 0.31 |
| **Q4 * Socio-economic level of family** |  | 6.80 (4) | 0.15 |
| **Q4 * Highest education level of either parent** |  | 0.38 (2) | 0.83 |
|  |  |  |  |
| **Q5 * Sex** | 3683.50 (-1.68 ) |  | 0.09 |
| **Q5 * Study Year** | 4077.50 (-0.73 ) |  | 0.46 |
| **Q5 * Permanent Address** |  | 2.25 (6) | 0.90 |
| **Q5 * Daily exercise/yoga/sports** | 5086.50 (-0.47 ) |  | 0.64 |
| **Q5 * Ethnicity** |  | 6.87 (5) | 0.23 |
| **Q5 * Strong faith in god** | 2800.00 (-0.05) |  | 0.96 |
| **Q5 * Socio-economic level of family** |  | 7.62 (4) | 0.11 |
| **Q5 * Highest education level of either parent** |  | 2.37 (2) | 0.31 |
|  |  |  |  |
| **Q6 * Sex** | 3684.50 (-1.67 ) |  | 0.09 |
| **Q6 * Study Year** | 4134.50 (-0.58 ) |  | 0.56 |
| **Q6 * Permanent Address** |  | 2.38 (6) | 0.88 |
| **Q6 * Daily exercise/yoga/sports** | 4079.00 (-2.93 ) |  | 0.003 |
| **Q6 * Ethnicity** |  | 5.15 (5) | 0.40 |
| **Q6 * Strong faith in god** | 2491.50 (-1.08 ) |  | 0.28 |
| **Q6 * Socio-economic level of family** |  | 7.82 (4) | 0.10 |
| **Q6 * Highest education level of either parent** |  | 2.10 (2) | 0.35 |
|  |  |  |  |
| **Q7 * Sex** | 3659.50 (-1.77 ) |  | 0.08 |
| **Q7 * Study Year** | 4074.50 (-0.75 ) |  | 0.45 |
| **Q7 * Permanent Address** |  | 7.39 (6) | 0.29 |
| **Q7 * Daily exercise/yoga/sports** | 5173.50 (-0.26) |  | 0.79 |
| **Q7 * Ethnicity** |  | 6.62 (5) | 0.25 |
| **Q7 * Strong faith in god** | 2657.00 (-0.54 ) |  | 0.59 |
| **Q7 * Socio-economic level of family** |  | 13.57 (4) | 0.01 |
| **Q7 * Highest education level of either parent** |  | 12.12 (2) | 0.002332 |
|  |  |  |  |
| **Q8 * Sex** | 3478.00 (-2.32 ) |  | 0.02 |
| **Q8 * Study Year** | 3661.50 (-1.92 ) |  | 0.05 |
| **Q8 * Permanent Address** |  | 4.26 (6) | 0.64 |
| **Q8 * Daily exercise/yoga/sports** | 5051.50 (-0.58) |  | 0.56 |
| **Q8 * Ethnicity** |  | 1.73 (5) | 0.88 |
| **Q8 * Strong faith in god** | 2787.50 (-0.10 ) |  | 0.92 |
| **Q8 * Socio-economic level of family** |  | 15.65 (4) | 0.003525 |
| **Q8 * Highest education level of either parent** |  | 2.81 (2) | 0.25 |
|  |  |  |  |
| **Q9 * Sex** | 3669.00 (-1.75 ) |  | 0.08 |
| **Q9 * Study Year** | 3945.00 (-1.11 ) |  | 0.27 |
| **Q9 * Permanent Address** |  | 5.81 (6) | 0.44 |
| **Q9 * Daily exercise/yoga/sports** | 5013.00 (-0.66) |  | 0.51 |
| **Q9 * Ethnicity** |  | 1.63 (5) | 0.90 |
| **Q9 * Strong faith in god** | 2583.50 (-0.79) |  | 0.43 |
| **Q9 * Socio-economic level of family** |  | 7.41 (4) | 0.12 |
| **Q9 * Highest education level of either parent** |  | 3.04 (2) | 0.22 |
|  |  |  |  |
| **Q10* Sex** | 3929.50 (-1.08 ) |  | 0.28 |
| **Q10 * Study Year** | 3999.00 (-1.01 ) |  | 0.31 |
| **Q10 * Permanent Address** |  | 2.94 (6) | 0.82 |
| **Q10 * Daily exercise/yoga/sports** | 4517.50 (-2.00 ) |  | 0.05 |
| **Q10 * Ethnicity** |  | 3.97 (5) | 0.55 |
| **Q10 * Strong faith in god** | 2367.00 (-1.61 ) |  | 0.11 |
| **Q10 * Socio-economic level of family** |  | 7.99 (4) | 0.09 |
| **Q10 * Highest education level of either parent** |  | 0.23 (2) | 0.89 |
|  |  |  |  |
| **Q11 * Sex** | 3867.50 (-1.18 ) |  | 0.24 |
| **Q11 * Study Year** | 3624.50 (-1.95 ) |  | 0.05 |
| **Q11 * Permanent Address** |  | 6.06 (6) | 0.42 |
| **Q11 * Daily exercise/yoga/sports** | 4647.00 (-1.55 ) |  | 0.12 |
| **Q11 * Ethnicity** |  | 7.05 (5) | 0.22 |
| **Q11 * Strong faith in god** | 2475.50 (-1.14 ) |  | 0.25 |
| **Q11 * Socio-economic level of family** |  | 5.92 (4) | 0.21 |
| **Q11 * Highest education level of either parent** |  | 0.60 (2) | 0.74 |
|  |  |  |  |
| **Q12 * Sex** | 3475.00 (-2.25 ) |  | 0.02 |
| **Q12 * Study Year** | 4168.00 (-0.49 ) |  | 0.62 |
| **Q12 * Permanent Address** |  | 11.32 (6) | 0.08 |
| **Q12 * Daily exercise/yoga/sports** | 5267.50 (-0.03) |  | 0.98 |
| **Q12 * Ethnicity** |  | 7.43 (5) | 0.19 |
| **Q12 * Strong faith in god** | 2563.50 (-0.85) |  | 0.40 |
| **Q12 * Socio-economic level of family** |  | 1.19 (4) | 0.88 |
| **Q12 * Highest education level of either parent** |  | 0.72 (2) | 0.70 |
|  |  |  |  |
| **Q13 * Sex** | 4066.50 (-0.65) |  | 0.51 |
| **Q13 * Study Year** | 4286.50 (-0.17) |  | 0.86 |
| **Q13 * Permanent Address** |  | 6.31 (6) | 0.39 |
| **Q13 * Daily exercise/yoga/sports** | 5173.00 (-0.27) |  | 0.79 |
| **Q13 * Ethnicity** |  | 11.31 (5) | 0.05 |
| **Q13 * Strong faith in god** | 2392.50 (-1.44 ) |  | 0.15 |
| **Q13 * Socio-economic level of family** |  | 8.17 (4) | 0.09 |
| **Q13 * Highest education level of either parent** |  | 0.79 (2) | 0.67 |
|  |  |  |  |
| **Q14 * Sex** | 3940.50 (-0.98) |  | 0.33 |
| **Q14 * Study Year** | 4345.50 (-0.01 ) |  | 0.99 |
| **Q14 * Permanent Address** |  | 7.03 (6) | 0.32 |
| **Q14 * Daily exercise/yoga/sports** | 4971.50 (-0.75 ) |  | 0.45 |
| **Q14 * Ethnicity** |  | 5.53 (5) | 0.35 |
| **Q14 * Strong faith in god** | 2086.00 (-2.43 ) |  | 0.02 |
| **Q14 * Socio-economic level of family** |  | 6.73 (4) | 0.15 |
| **Q14 * Highest education level of either parent** |  | 0.71 (2) | 0.70 |
|  |  |  |  |
| **Q15 * Sex** | 3995.50 (-0.83 ) |  | 0.41 |
| **Q15 * Study Year** | 4348.00 (-0.01 ) |  | 1 |
| **Q15 * Permanent Address** |  | 12.24 (6) | 0.06 |
| **Q15 * Daily exercise/yoga/sports** | 4752.00 (-1.28 ) |  | 0.20 |
| **Q15 * Ethnicity** |  | 0.86 (5) | 0.97 |
| **Q15 * Strong faith in god** | 2675.00 (-0.47) |  | 0.64 |
| **Q15 * Socio-economic level of family** |  | 3.17 (4) | 0.53 |
| **Q15 * Highest education level of either parent** |  | 0.61 (2) | 0.74 |
|  |  |  |  |
| **Q16 * Sex** | 4178.50 (-0.34) |  | 0.74 |
| **Q16 * Study Year** | 3631.00 (-1.92 ) |  | 0.05 |
| **Q16 * Permanent Address** |  | 0.52 (6) | 1 |
| **Q16 * Daily exercise/yoga/sports** | 4922.00 (-0.87 ) |  | 0.39 |
| **Q16 * Ethnicity** |  | 5.61 (5) | 0.35 |
| **Q16 * Strong faith in god** | 2600.00 (-0.72) |  | 0.47 |
| **Q16 * Socio-economic level of family** |  | 3.50 (4) | 0.48 |
| **Q16 * Highest education level of either parent** |  | 4.55 (2) | 0.10 |
|  |  |  |  |
| **Q17 * Sex** | 4158.50 (-0.44 ) |  | 0.66 |
| **Q17 * Study Year** | 3580.50 (-2.30 ) |  | 0.02 |
| **Q17 * Permanent Address** |  | 2.62 (6) | 0.86 |
| **Q17 * Daily exercise/yoga/sports** | 4949.00 (-0.90) |  | 0.37 |
| **Q17 * Ethnicity** |  | 10.21 (5) | 0.07 |
| **Q17 * Strong faith in god** | 2642.50 (-0.65) |  | 0.52 |
| **Q17 * Socio-economic level of family** |  | 10.09 (4) | 0.04 |
| **Q17 * Highest education level of either parent** |  | 3.88 (2) | 0.14 |
|  |  |  |  |
| **Q18 * Sex** | 3844.50 (-1.32 ) |  | 0.19 |
| **Q18 * Study Year** | 3436.00 (-2.62 ) |  | 0.01 |
| **Q18 * Permanent Address** |  | 12.36 (6) | 0.05 |
| **Q18 * Daily exercise/yoga/sports** | 4710.50 (-1.48 ) |  | 0.14 |
| **Q18 * Ethnicity** |  | 2.41 (5) | 0.79 |
| **Q18 * Strong faith in god** | 2703.50 (-0.40) |  | 0.69 |
| **Q18 * Socio-economic level of family** |  | 3.62 (4) | 0.46 |
| **Q18 * Highest education level of either parent** |  | 3.83 (2) | 0.15 |
|  |  |  |  |
| **Q19 * Sex** | 3442.00 (-2.35 ) |  | 0.02 |
| **Q19 * Study Year** | 2873.00 (-4.00 ) |  | 0.00006 |
| **Q19 * Permanent Address** |  | 4.93 (6) | 0.55 |
| **Q19 * Daily exercise/yoga/sports** | 4869.00 (-1.01 ) |  | 0.31 |
| **Q19 * Ethnicity** |  | 5.06 (5) | 0.41 |
| **Q19 * Strong faith in god** | 2606.50 (-0.71) |  | 0.48 |
| **Q19 * Socio-economic level of family** |  | 2.60 (4) | 0.63 |
| **Q19 * Highest education level of either parent** |  | 2.14 (2) | 0.34 |
|  |  |  |  |
| **Q20 * Sex** | 3207.00 (-3.00 ) |  | 0.002 |
| **Q20 * Study Year** | 3594.00 (-2.05 ) |  | 0.04 |
| **Q20 * Permanent Address** |  | 1.76 (6) | 0.94 |
| **Q20 * Daily exercise/yoga/sports** | 5116.50 (-0.40) |  | 0.69 |
| **Q20 * Ethnicity** |  | 4.42 (5) | 0.49 |
| **Q20 * Strong faith in god** | 2615.00 (-0.68) |  | 0.50 |
| **Q20 * Socio-economic level of family** |  | 6.94 (4) | 0.14 |
| **Q20 * Highest education level of either parent** |  | 1.25 (2) | 0.53 |

*Abbreviation:- Q: Question, df: Degrees of freedom*
